# Role of Natural Binding Proteins in Therapy and Diagnostics

**DOI:** 10.3390/life14050630

**Published:** 2024-05-15

**Authors:** Marco Eigenfeld, Kilian F. M. Lupp, Sebastian P. Schwaminger

**Affiliations:** 1 Otto-Loewi Research Center, Division of Medicinal Chemistry, Medical University of Graz, Neue Stiftingtalstraße 6, 8010 Graz, Austria; 2 BioTechMed-Graz, Mozartgasse 12/II, 8010 Graz, Austria

**Keywords:** binding proteins, nanoparticles, binding domains, personalized medicine, drug delivery, targeted delivery, diagnostics

## Abstract

This review systematically investigates the critical role of natural binding proteins (NBPs), encompassing DNA-, RNA-, carbohydrate-, fatty acid-, and chitin-binding proteins, in the realms of oncology and diagnostics. In an era where cancer continues to pose significant challenges to healthcare systems worldwide, the innovative exploration of NBPs offers a promising frontier for advancing both the diagnostic accuracy and therapeutic efficacy of cancer management strategies. This manuscript provides an in-depth examination of the unique mechanisms by which NBPs interact with specific molecular targets, highlighting their potential to revolutionize cancer diagnostics and therapy. Furthermore, it discusses the burgeoning research on aptamers, demonstrating their utility as ‘nucleic acid antibodies’ for targeted therapy and precision diagnostics. Despite the promising applications of NBPs and aptamers in enhancing early cancer detection and developing personalized treatment protocols, this review identifies a critical knowledge gap: the need for comprehensive studies to understand the diverse functionalities and therapeutic potentials of NBPs across different cancer types and diagnostic scenarios. By bridging this gap, this manuscript underscores the importance of NBPs and aptamers in paving the way for next-generation diagnostics and targeted cancer treatments.

## 1. Introduction

The landscape of cancer in the United States in 2023 depicts a daunting scenario, marked by an estimated 1,958,310 new cancer cases and 609,820 cancer-related deaths. For instance, prostate cancer among men has increased with an annual growth of 3% since 2014, accounting for about 99,000 new cases each year [1]. However, breast cancer emerges as the globally most prevalent cancer among women, showing an ongoing upward trend [2,3]. The effective treatment of breast cancer is influenced by several factors, including the stage of the disease, tumor aggressiveness, individual response to treatment, and lifestyle choices like medication, smoking, or alcohol consumption [3]. Despite these challenges, the rate of lung cancer has been declining more slowly in women than in men, with a yearly decrease of 1.1% in women compared to 2.6% in men between 2015 and 2019 [1]. However, the continuous decline in the cancer death rate, including a 1.5% reduction from 2019 to 2020, contributes to a 33% overall reduction since 1991, highlighting the significant impact of advancements in treatment across various cancer types.

By testing 1000 women who are 50 years old every year for a decade, it is possible to prevent one death from breast cancer [4]. This underscores the potential life-saving benefits of early detection in managing breast cancer.

Traditional long-term cancer treatments, such as chemotherapy, are associated with significant side effects, including hair loss, skin issues, hot flashes, and nausea [5,6]. They can, furthermore, include fertility issues and heart and lung problems [5,6]. Breast surgery as a consequence of breast cancer treatment and mastectomy have physical and psychological impacts, such as changes in body image and loss of breast sensitivity [6]. Additionally, some breast cancers may develop drug resistance, necessitating alternative treatment approaches [5].

However, significant progress has been made recently in developing combination therapies for breast cancer. These include monoclonal antibodies (mAbs) like trastuzumab, pertuzumab, and margetuximab, combined with cytostatic drugs and tyrosine kinase inhibitors (TKIs) in regimens such as the Cleopatra regime [7]. The introduction of mAbs has notably improved early-stage breast cancer treatment outcomes, though only a minority of patients respond positively to initial treatment, leading to a poor prognosis in advanced stages [8,9]. Metastasis and resistance development often result in low cure rates and limited survival. The SOPHIA study showed that margetuximab combined with chemotherapeutics offered no significant overall survival benefit compared to trastuzumab but could be an alternative for patients not responding to the Cleopatra regime [10].

In oncology, significant advancements have also been made in the development of prophylactic vaccines, especially against cervical cancer, which is often linked to persistent high-risk human papillomavirus (HPV) infections. Current prophylactic vaccines are effective against 90% of HPV infections but offer limited benefits for preexisting infections, underscoring the need for therapeutic vaccine development [11].

Moreover, in the field of biomedical applications, significant advancements have been achieved through the integration of nanoparticle usage in combination with biological systems. Nanoparticles have gained attention for their potential in medical applications, particularly drug delivery and therefore cancer treatment. Organic nanoparticles are valued for their unique properties but face challenges like potential toxicity, instability, and limited in vivo circulation [12,13,14,15].

To enhance the therapeutic outcomes of nanoparticle-mediated drug delivery, it is crucial to develop carriers that not only release drugs in a controlled manner but also efficiently navigate through and overcome the body’s complex biological barriers [16,17]. Overcoming these barriers significantly contributes to the nanoparticles’ therapeutic effectiveness, which refers to their ability to improve health outcomes by delivering drugs more efficiently to the target site, thereby maximizing the therapeutic impact while minimizing side effects [17,18]. In this context, protein-based nanoparticles stand out as a particularly promising carrier option due to their biocompatibility, biodegradability, and relatively low potential to elicit immune responses [18,19]. These nanoparticles can be engineered to respond to specific physiological stimuli—such as pH changes in tumor cells, temperature shifts, or enzymatic activity—enabling them to cross biological barriers more effectively and release their drug payload precisely where needed [17,18]. The mild and nontoxic conditions required for their preparation further underscore their suitability for therapeutic applications [19,20,21,22]. Thus, by leveraging the unique properties of protein-based nanoparticles, it is possible to achieve a higher level of control over drug release kinetics and targeting, directly linking the design and functionalization of these carriers to improved therapeutic outcomes.

## 2. Proteins in Conjunction with Nanoparticles

In the realm of biomedical applications, the fusion of nanotechnology with biological systems has led to remarkable advancements, particularly in the development of sophisticated drug delivery systems. One significant breakthrough in this area is the advent of lipid-based nanoparticles, such as liposomes, which have already made their way into clinical use [16]. For instance, liposomal formulations like Doxil^®^, the liposome-encapsulated doxorubicin, represent a milestone in chemotherapy, offering targeted delivery that reduces toxicity and improves efficacy in cancer treatment [23]. This innovation exemplifies how nanotechnology can revolutionize therapeutic strategies by enhancing the delivery and bioavailability of drugs. Building on such foundational advancements, researchers are now exploring even more innovative approaches, including the design of stimuli-responsive nanoparticles that can release their payload in response to specific triggers within the body, as mentioned earlier. These cutting-edge developments signify a new era in personalized medicine, where drug delivery systems are not only more efficient but also finely tuned to the patient’s unique physiological conditions.

An innovative method, magnetically controlled drug delivery, leverages drug-loaded magnetic nanoparticles (MNPs) to precisely target affected areas through an external magnetic field. These MNPs possess a large specific surface area, allowing them to transport significant drug doses directly to the target site, thus achieving high local drug concentrations [24,25]. Their growing recognition in the medical field stems from their advantageous characteristics, including small size, cost efficiency, and adaptability in production and modification, which make them valuable for both diagnostic and therapeutic uses [26,27,28].

Iron oxide nanoparticles (IONs) are widely utilized as contrast agents for T1- and T2-weighted magnetic resonance imaging (MRI) in clinical diagnosis [29,30]. IONs are particularly studied for their role as a T2 contrast agent in MRI because they efficiently shorten transverse relaxation times [31]. Notably, IONs have demonstrated several beneficial properties, including long blood half-lives, low toxicity, and flexible surface chemistry [32,33].

**Figure 1:**
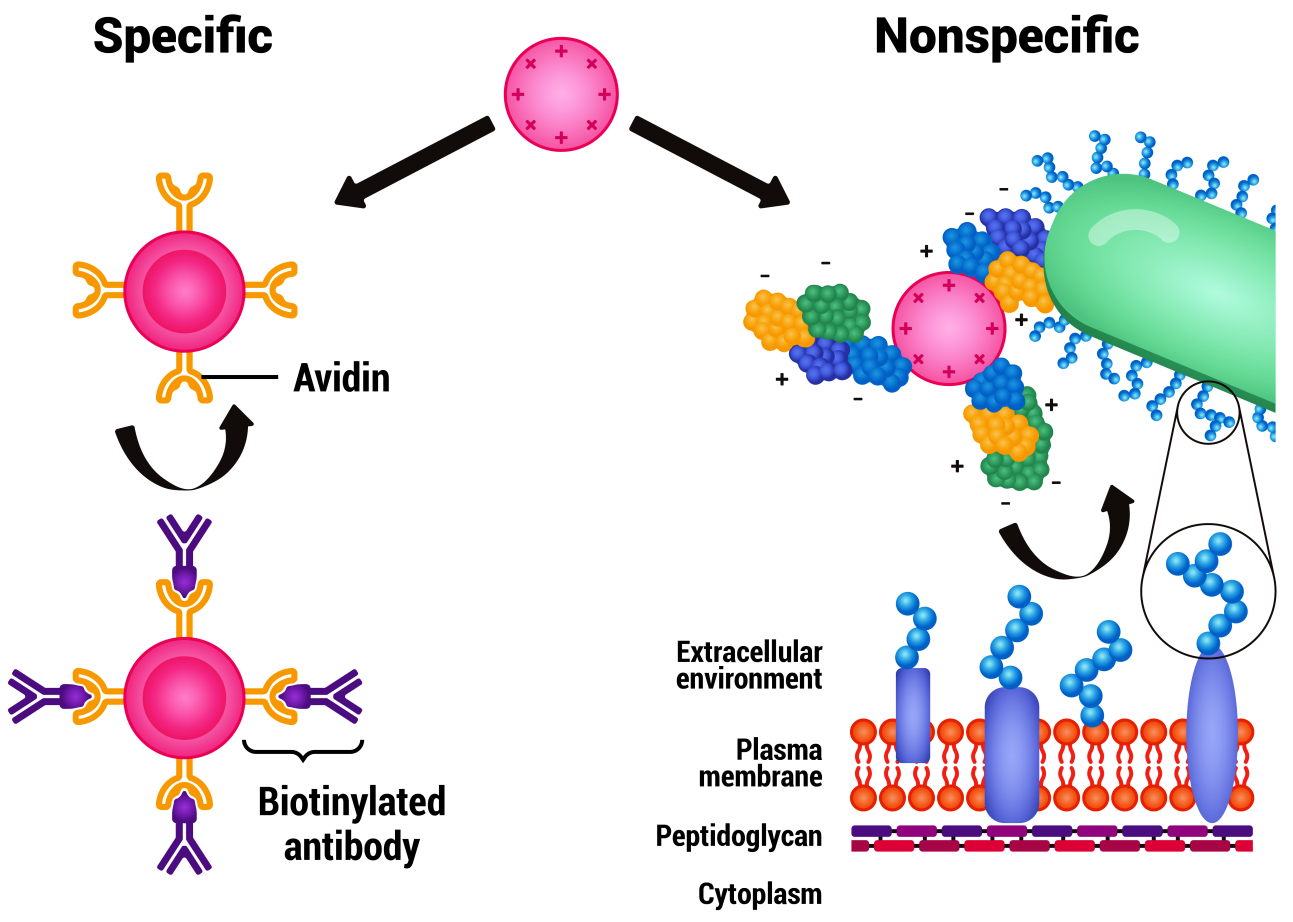
An illustrative comparison of particle modification techniques for selective orientation in catalytic processes on the left side; on the right, the indiscriminate attachment of cells due to interactions between cell wall proteins and surface charges.

Furthermore, the combination of proteins with nanoparticles is a key example of this technological integration. Proteins or antibodies, with their surface functional groups, allow for easy modification of nanoparticle surfaces as indicated in Figure 1. This characteristic is highly beneficial for targeted drug delivery, diagnostics, and tissue engineering applications [34,35]. Additionally, the hollow structure of certain proteins facilitates the encapsulation of small-molecule drugs or metal nanoparticles, enhancing the potential for drug delivery and combination therapy [36,37].

Nanoparticles, particularly MNPs, are versatile in tumor treatment, with two distinct approaches: (i) conjugating specific antibodies to MNPs for selective binding to receptors and inhibition of tumor growth through drugs, loaded on the particles, resulting in targeted therapy; (ii) employing targeted MNPs for hyperthermia in tumor therapy [38]. These approaches exemplify how nanotechnology enhances the precision and efficacy of biomedical treatments. Beyond liposomal drugs, several nanoparticle-based formulations have gained approval for clinical use, such as Eligard^®^ for prostate cancer in the USA [39] and Nanoxel^®^ for various cancers in India [40]. Additionally, the European Medicines Agency (EMA) has approved Apealea for ovarian cancers [41] and NanoTherm for glioblastoma and other cancers [42], highlighting nanotechnology’s expanding role in approved cancer therapies and approaches. Tests on superparamagnetic iron oxide nanoparticles have also been conducted to track their migration as a magnetic tracer, showing an increase in monitoring counts on the skin’s surface [43].

There exists a variety of proteins suitable for the creation of protein-based nanoparticles, with many being producible through recombinant protein synthesis. Commonly used proteins include fibroin, human serum albumin, gliadin, lipoproteins, and elastin-like polypeptides, as detailed in reviews by Hong et al. [44], Jain et al. [19], and Yao et al. [45]. This chapter focuses on biomimetic materials and natural binding proteins, areas yet to be extensively reviewed and of particular interest in this discussion.

### 2.1. Various Nanoparticles

In the landscape of advancing biomedical technologies, it is crucial to distinguish between biomimetic nanoparticles and nanoparticles with immobilized proteins, while both are integral to the realm of drug delivery, they exhibit distinct characteristics and functionalities.

#### 2.1.1. Biomimetic Materials

Biomimetic nanoparticles are engineered to mimic biological surfaces, like cells or viruses, in both form and function. This mimicry extends beyond mere structural imitation; it encompasses the replication of biological behaviors and interactions. Therefore, one should differentiate between biomimetic materials/nanoparticles and protein-based biomaterials. Biomimetic materials are an integral part of generating natural mimicry. These nanoparticles are made from a variety of materials, including metals, polymers, lipids, and even composite materials. By emulating not only the chemical composition and structure but also the biological characteristics and functions of natural materials, this approach is instrumental in creating efficient drug delivery systems capable of navigating biological barriers and utilizing cellular recognition and uptake mechanisms [46]. Due to their programmable chemistry and biocompatibility, biomimetic materials have found applications in innovative medical technologies, such as tendon-driven myoelectric soft hand exoskeletons [47,48], biomimetic scaffolds for tendon regeneration [49], cartilage-lubricating polymers [50], and in dentistry [51].

Specifically, apatite nanocrystals [52] and biomimetic $Fe_{3}O_{4}$ nanoparticles coated with red blood cell (RBC) membranes demonstrate the capacity for targeted and controlled drug delivery, with the latter showing prolonged circulation time [53], immune response evasion [54], and immunomodulatory effects [53] resembling artificial antigen-presenting cells (APCs) [54]. These characteristics highlight the potential of biomimetic nanoparticles in regulating immune responses, ensuring long-term circulation and achieving high target specificity.

The ongoing debate around the definition of biocompatibility, as discussed by Naahidi et al. [55], underscores the absence of standardized criteria for evaluating nanoparticle safety in drug delivery systems. This lack of clear guidelines highlights the need for safety assessments of nanoparticles’ impacts on human health, considering their dynamic physicochemical properties and the importance of understanding their biological interactions [55]. The capability of nanoparticles to provide targeted therapy, minimizing side effects while maximizing efficacy, points to their significant potential in revolutionizing drug delivery and diagnostic methods, further emphasizing the importance of research on nanoparticle biocompatibility and mechanisms of action [55].

The second class are protein-based biomaterials, as outlined in the review by Zhang et al. (2023) [56], which exemplify this integration in medical applications. These biomaterials are noted for their “encoded and programmable mechanical properties such as superelasticity, plasticity, shape adaptability, and excellent interfacial behavior, derived from sequence-guided backbone structures” [56]. These materials are primarily made from proteins, which can be sourced from animals or plants. Despite several advancements, the traditional method of regenerating protein materials from natural sources faces issues like low yield and structural damage due to extraction process steps. As highlighted by Lavickova et al. [57], the concentration of DNA templates used for the regeneration of specific proteins plays a significant role in achieving optimal regeneration efficiency. Therefore, developing alternative strategies for fabricating protein materials, like membrane proteins, is crucial [58]. A promising approach is the heterologous expression of natural proteins with a modular assembly approach, involving the creation of standardized, easy-to-assemble protein modules with specific structures and functions [59].

A notable example within this area are “virus-like particles” (VLPs), which are protein-based nanoparticles formed by the self-assembly of viral coat proteins [60,61,62]. These nanoparticles mimic natural viruses in structure but are safe for human use as they lack viral nucleic acids, thus preventing replication and viral infection [63]. However, their resemblance to viruses can potentially activate the immune system [60,64], a challenge that various research projects are addressing through different production hosts like plant [63], yeast [65], or insect cells [62].

In summary, the main difference between both parts of biomimetic materials is their function and utilization. Biomimetic nanoparticles focus on mimicking specific biological functions at the nanoscale for targeted therapies, whereas protein-based biomaterials focus on exploiting the inherent properties of proteins for the applications. In the next section, it is explained how nanoparticles with immobilized natural binding proteins leverage the specificity of protein functions to achieve targeting and interaction within the body, reflecting a more focused approach in biomedical applications.

#### 2.1.2. Natural Binding Proteins

NBPs, including those that attach to DNA and RNA, play a crucial role in both cancer development and treatment, as noted in recent studies [66,67]. Future research will also look into fat- and sugar-binding proteins, which have unique sections known as fatty acid- or carbohydrate-binding domains [68,69]. These proteins are found in various organisms, such as the bacteria *Bacillus circulans* and fungi like *Trichoderma* species. They are remarkable for their ability to specifically and strongly attach to certain molecules, including fats, chitin, chitosan, and cellulose [70,71,72]. DNA- and RNA-binding proteins are essential in all forms of life, helping control gene activity by acting as switches that turn genes on or off [73]. This regulation is crucial for making proteins correctly and responding to changes in the environment [73]. Having introduced the pivotal role of natural binding proteins (NBPs) in cancer development and treatment, we now delve deeper into their specific functionalities and mechanisms, which underscore their dual utility in both suppressing tumors and enhancing drug delivery.

NBPs play a pivotal role in cancer therapy, exhibiting dual functionality by both suppressing tumors and enhancing the targeted delivery of drugs with their highly specific binding abilities. These proteins can indirectly influence tumor growth and progression through various mechanisms. They might block interactions between tumors and their surrounding microenvironment, inhibit angiogenesis as noted by Smith [74], or direct the immune system to target and destroy tumor cells. This multifaceted approach not only underscores the importance of NBPs in devising precise treatment strategies but also highlights their utility in diagnostics and therapeutic applications, where their selective binding properties are leveraged for targeted treatments and diagnostic procedures. Beyond their direct impact on tumor growth and interaction, NBPs’ unique capabilities extend to innovative applications in targeted drug delivery and diagnostics. This is exemplified through their precision in attaching to specific molecular targets, a principle that is foundational to advancing cancer therapy.

Furthermore, a specific application of NBPs in targeted drug delivery involves the use of recombinant proteins that possess, for example, either a C- or N-terminal chitin-binding domain. These binding proteins can specifically attach to inert chitin particles [71,75,76], allowing for oriented immobilization. This method is particularly compatible with the human bloodstream, which naturally lacks chitin-like compounds. While targeted drug delivery showcases the therapeutic potential of NBPs, their role is not confined to treatment alone. The following discussion explores how NBPs contribute to the immune system’s response to cancer and serve as powerful tools in diagnostics and prognosis, highlighting their versatility in oncology.

In contrast, substances like chitin and chitosan are known to stimulate cytokine production by activation of transcription factors like NF-*κ*B and AP-1, draw leukocytes, and differently activate macrophages [77,78,79], showcasing a different mechanism by which NBPs can contribute to the immune system’s response to cancer.

NBPs also serve as tumor markers, aiding in diagnostics and prognosis, like prostate-specific antigen (PSA) used for prostate cancer screening [80]. Their specificity makes them ideal candidates for targeted drug development, aligning with personalized medicine trends in oncology [45]. Additionally, NBPs’ influence on the tumor microenvironment provides insights into cancer progression and new treatment strategies.

**Figure 2:**
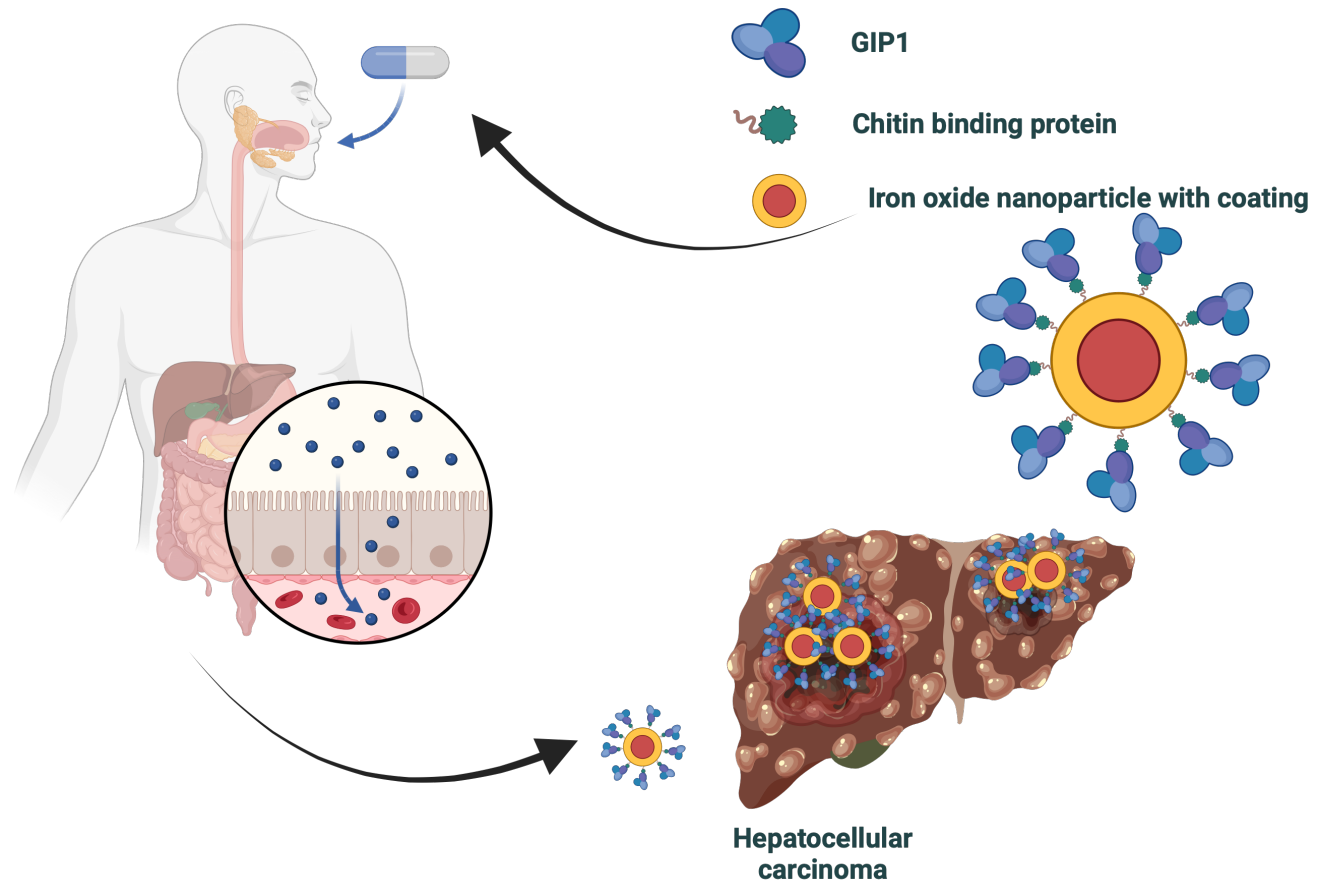
Schematic diagram of an application scenario for the use of natural binding proteins in targeted drug delivery and MRI targeting. Created using biorender.com.

A promising technique for detecting liver cancer uses nanoparticles paired with a special protein linker, illustrated in Figure 2. This linker has two main functions: it connects to the nanoparticles through a chitin-binding domain, and it targets liver cancer cells that express a specific protein, glypican-3 (GPC3) [81]. GPC3 is often found in high amounts on liver cancer cells but is rare in healthy liver tissue.

In this method, nanoparticles carrying this dual-function protein linker act as enhanced contrast agents in medical imaging. When injected into a patient, the GIP1 part of the linker specifically binds to GPC3 on the liver cancer cells. This targeted binding leads the nanoparticles to accumulate precisely at the tumor site. To illustrate the practical impact of NBPs in oncology, let us examine a case study focusing on liver cancer detection. This example demonstrates how NBPs, when integrated with advanced nanoparticle technology, can revolutionize cancer diagnostics by improving accuracy and specificity.

The key to this approach is the dual ability of the protein linker. It can both adhere to the chitin on the nanoparticles and latch onto GPC3 on the liver cancer cells. This dual action improves the performance of the nanoparticles as imaging agents and increases the accuracy of tumor detection in scans.

Using this targeted approach for contrast agents results in more precise and detailed imaging. This is vital for the early detection of liver cancer, accurately determining the tumor’s size and location. The deployment of GIP1, efficiently produced in *Escherichia coli* cells as indicated by Janski et al. [82], marks a significant step forward in liver cancer diagnostic imaging.

In the treatment phase, the nanoparticles are loaded with chemotherapy drugs or other effective medications. Postinjection, these complexes bind specifically to liver cancer cells, releasing the drug right at the target site. This targeted approach allows for a higher concentration of the drug at the tumor site, sparing healthy tissue. It enhances treatment efficacy and simultaneously reduces side effects.

This method represents a novel approach to targeted drug delivery, illustrating how NBPs can be utilized. Due to their specific attachment to nanoparticles, they enable more effective and less invasive cancer therapies.

In summary, the integration of natural binding domains represents a promising direction in drug delivery technology. These novel platforms aim to overcome existing limitations and revolutionize drug delivery. This chapter underscores the importance of bio-inspired design and advanced material engineering in developing effective drug delivery systems that navigate the complexities of biological systems and optimize therapeutic outcomes.

## 3. Exploring the Role of Binding Domains in Cancer Treatment: Applications, Innovations, and Impact in Oncology

The current landscape of oncology is experiencing a significant paradigm shift, largely propelled by the advancements in antibody–drug conjugates (ADCs) [83], which epitomize the exploration and application of novel approaches. This evolution is characterized by a strategic transition towards highly targeted cancer therapies that promise enhanced efficacy and minimized toxicity, a leap forward from the constraints of traditional chemotherapy [83]. This shift is quantitatively evident in the deployment of medications, notably through a significant reduction in chemotherapy treatments, such as a 20% decrease in its use for breast cancer due to genomic testing [84].

Exploring further becomes possible through the use of binding domains found in NBPs. NBPs are distinguished by their remarkable capability to bind specifically to certain molecules. The integration of ADCs with NBP motifs is heralding a new era in the realm of oncology, offering a promising pathway toward the realization of highly specific and efficacious cancer therapies and diagnostics. This innovative approach not only leverages the precision targeting capabilities of monoclonal antibodies inherent in ADCs but also enhances therapeutic outcomes through the synergistic combination with diverse NBP motifs.

**Figure 3:**
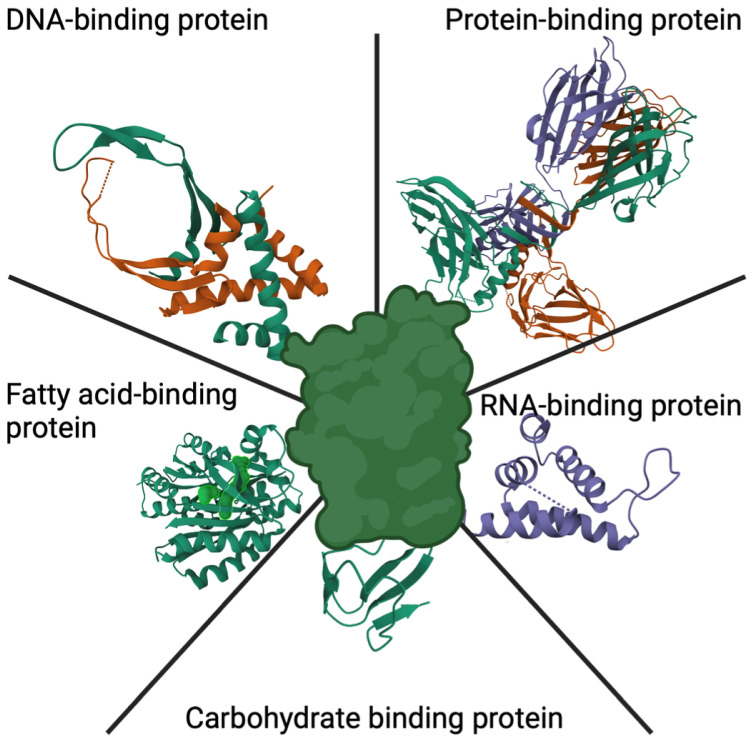
Diagram illustrating various binding proteins and motifs within the complete protein complex. Data of the protein structure were obtained from www.rcsb.org (datasets 2BSD, 1ED7, 3FYS, 3RHI, and 2W4S; accessed on 2 May 2024). Created using biorender.com.

The following section is dedicated to examining the binding domains in NBPs shown in Figure 3, their natural occurrence, and their significant roles. It highlights the various applications they have in cancer treatment, the innovative approaches being developed around them, and the extensive impact they are making in the field of oncology. Furthermore, the utilization of these binding domains across different facets of cancer therapy are explored, including the domains’ use in targeted drug delivery systems, the development of novel diagnostic tools, and their potential to fundamentally transform cancer treatment methodologies.

### 3.1. DNA-Binding Domains

DNA–protein interactions are crucial for regulatory proteins, which recognize specific DNA sequences of 8–20 base pairs amidst millions, guiding the protein to its functional area [85]. DNA-binding domains (DBDs) on the other hand, are crucial molecular components that enable proteins, particularly transcription factors, to interact specifically with DNA. These domains have evolved to recognize and bind to specific DNA sequences, displaying diverse structural features, including α-helices, β-sheets, and disordered regions [86]. These structures, such as helices and loops, interact with DNA’s grooves and turns to identify specific sequences [86]. What distinguishes these proteins from others is primarily their ability to specifically identify DNA sequences among the vast expanse of the genome, enabling precise regulation of gene expression. This specificity is achieved through the combination of structural motifs within the protein that match the unique shape and chemical properties of target DNA sequences. For instance, the helix–turn–helix motif, commonly found in these proteins, allows for snug fitting into the DNA major groove, where it can make specific contacts with the bases [87]. Similarly, zinc finger motifs use a combination of alpha helices and beta sheets stabilized by zinc ions to recognize specific DNA sequences [88]. In contrast, other proteins might interact with DNA in a more generalized manner, lacking the fine-tuned specificity of these binding domains. These might include histone chaperones, which effectively prevent nonspecific contacts between the negatively charged DNA and the positively charged histones, ensuring an orderly assembly of the nucleosome structure [89], or enzymes like DNA polymerase, which reads the DNA template but does not have the sequence-specific binding properties of transcription factors or other DNA-binding proteins discussed here. The target search of DNA polymerase is dominated by transient nonspecific DNA binding [90]. The mobility of these proteins during their target search is dictated by DNA interactions rather than their molecular weights [90]. Specifically, DNA-binding proteins, regardless of their size, concentration, or function, spend the majority (58–99%) of their search time bound to DNA, indicating that transient DNA-binding events dominate the target search process [90].

#### 3.1.1. Classification, Characteristics, and Function

DBDs are typically categorized based on their structural characteristics. As a consequence, transcription factors (TFs) are grouped into families according to the type of DBD they contain. In general, large domain databases classify protein domains hierarchically; while the *class* reflects the three-dimensional structure, the architecture describes the arrangement of secondary structures (Table 1). A *superfamily* is a protein group with common evolutionary origin, and the *family* has clear evolutionary relationships [91]. However, the literature does not appear to have adopted this structure. Regarding DBDs, a distinction is made between five different superclasses of domains: basic domains, zinc-coordinating DNA-binding domains, helix–turn–helix, beta scaffold factors with minor groove contacts, and other transcription factors (indicated in Table 1). Each superclass consists of several classes; for example, leucine zipper factors, helix–loop–helix factors, and their combinations, are classes of basic domain superclass [86]. Generally, transcription factors within the same family show similar DNA-binding specificities, although variations can occur due to changes in specific amino acids within the DBD [92].

One of the defining characteristics of DBDs is their modular nature, allowing them to be isolated from the rest of the transcription factor without loss of function and therefore allowing the study of multiple effects [94]. This modularity is advantageous for structural studies, facilitating techniques like crystallization or nuclear magnetic resonance (NMR) spectroscopy. Hence, the structures of DNA-binding domains alone or combined with DNA can be easily observed [86].

A prominent example of a protein with distinct DBDs is the Epstein–Barr virus nuclear antigen 1 (EBNA1). EBNA1’s DNA-binding region consists of two different domains: the C-terminal (core domain, residues 504–607 [95]) and the N-terminal (flanking domain, residues XY-YZ) [96]. The flanking domain is unique to EBNA1 and crucial for sequence-specific binding. This domain attaches to the outer portion of the EBNA1-binding site, while the core domain connects to the inner portion [96]. Interestingly, the core domain structurally resembles the DNA-binding and dimerization domain of the E2 protein from bovine papilloma virus, indicating also a role in sequence-specific DNA binding. This structural resemblance is notable given the lack of sequence similarity and known evolutionary links between the EBNA1 (herpes) and E2 (papovavirus) virus families [96].

Certain types of DNA-binding domains stand out due to their ubiquity and functional relevance. For example, the superclass of zinc-coordinating DNA-binding domains utilizes a zinc atom, often coordinated by cysteine and histidine residues, to recognize three to four bases of DNA [97,98]. This domain is frequently found in transcription factors like Sp1 [99]. Studying the superclass of helix–turn–helix (HTH) transcription factors can provide further insight into DBD functionality. X-ray crystallography has revealed their surface structure, including a short α-helix known as the recognition helix, predicted to fit partially within DNA’s major groove [87]. This structural feature enables specific interactions between residues and DNA bases, crucial for sequence-specific DNA binding, as observed in proteins like the cyclic AMP receptor protein (CRP) of *E. coli*, the bacteriophage λ regulatory protein Cro, and the NH2-terminal domain of λ repressor [97]. The typical dissociation constants of DNA-binding proteins are in the mid- to lower molar range [100,101] (130–1000 nM), indicating a very high affinity.

#### 3.1.2. The Role of DBDs in Oncology and Applications

Transcription factors, crucial in oncology due to their DNA-binding roles, become potential drug targets when mutated or dysregulated, leading to cancer by disrupting gene expression, including pathways for cell differentiation and death [102,103,104]. Targeting transcription factor activity has shown promise both preclinically and clinically through strategies like inhibiting protein interactions, DNA binding, and modulating degradation processes [105]. Innovations including modulation of auto-inhibition, use of proteolysis targeting chimeras (PROTACs), and combination therapies aim to refine cancer treatment by targeting these transcription factors’ unique properties [106].

Enhancers are regulatory parts of DNA that are involved in controlling which genes are turned on in different body tissues. New research indicates that point mutations in these enhancers, or in elements that help enhancers communicate with other parts of DNA, can lead to cancers that specifically affect certain tissues [106].

One of the key approaches in developing cancer therapeutics involves targeting the specific interactions between DBDs and DNA. This targeted approach is pivotal in enhancing the efficacy of cancer therapy because it directly interferes with the functioning of potent oncogenic transcription factors [107]. One example is FOXM1, a transcription factor crucial for cancer initiation, progression, and drug resistance, and its regulatory network, which is therefore a major predictor of adverse outcomes in various human cancers [107]. Furthermore, high-throughput screening methods have been instrumental in identifying and selectively inhibiting DNA-binding proteins [108]. Additionally, the study of proteins like Smad4, a TGF-β-inducible DNA-binding protein, underscores the importance of these proteins in understanding cancer biology and devising treatment strategies [109]. Smad4’s involvement in TGF-β signaling pathways highlights the intricate relationship between growth factors and gene regulation in the development of cancer [109]. The advancement of such recent therapeutic strategies represents a significant development [108].

Beyond the scope of oncology, DBDs play a crucial role in molecular biology and biotechnology. Customized DBDs can be used to manipulate DNA in a sequence-specific manner, a principle integral to technologies such as CRISPR/Cas9 [110]. By coupling DBDs with transcriptional modulators, researchers can regulate gene expression, providing valuable insights into cellular pathways [110].

In the context of DNA-binding drugs, examples such as amsacrine demonstrate their effectiveness in treating acute lymphoblastic leukemia by targeting DNA topoisomerase II [111]. The development of DACA (N-[2-(dimethylamino)ethyl]acridine-4-carboxamide) for lung adenocarcinoma [112], along with its derivatives like SN 28049, illustrates the evolving landscape of DNA-binding drugs in cancer therapy [113]. While these drugs act by disrupting topoisomerase II, an enzyme that helps manage the structure of DNA during cell division, there remain challenges in pharmacokinetics and toxicity because amsacrine can also affect normal, healthy cells that divide rapidly [114]. Future research is geared towards understanding the interplay between DNA-binding drugs, topoisomerase, and the immune system, with the aim of improving cancer treatment strategies by preventing harm to healthy cells.

### 3.2. Protein-Binding Domains

In the complex landscape of cellular biology, proteins rarely operate independently. Instead, they engage in intricate networks of interactions, which are crucial in a multitude of cellular functions. This section delves into protein–protein interaction (PPI) domains, specialized regions that enable such interactions with high specificity [115].

#### 3.2.1. Characteristics and Functions of PPI Domains

PPI domains facilitate the precise and selective interaction between proteins. They act as specialized docking stations, allowing proteins to recognize and bind to each other. PPI domains are fundamental to mechanisms such as signal transduction pathways, cellular trafficking, DNA replication, and cell-cycle control [1]. Many of these processes involve a protein domain binding to a short sequence (3–10 amino acids) of another protein characterized by a specific pattern [116]. For instance, the POZ (pox virus and zinc finger) domain [117], or the BTB/POZ domain found in genes of DNA viruses [118], exemplifies this binding specificity. While zinc finger domains are predominantly recognized for their DNA-binding abilities and role in transcription factors [97], they also possess the capacity to bind to protein sequences [119]. In general, PPI domains are integral in ensuring that cellular processes are conducted with precision, specificity, and coordination [115]. A more detailed holistic classification of these domains is not available from the literature. The determined affinities of PPIs vary in ranges between 100 and 3000 nM (Table 4). Due to the sheer diversity and size of protein–protein bonds, mapping and classifying the domains is a challenge [120]. While rule-based algorithms allow for some classification of PPIs, the specific classification process may vary depending on the algorithm used [121]. Therefore, many different approaches for classification, based on machine learning, have been published. For example, Urquiza et al. found the eight important features for the prediction of PPIs, which were validated by a ROC analysis [122]. A web server called Protein Complex Prediction by Interface Properties (PCPIP) is provided by Subhrangshu and Saikat [120], which can predict whether the interface of a given protein–protein dimer complex resembles known protein interfaces. The server is freely available at http://www.hpppi.iicb.res.in/pcpip/ (accessed on 29 March 2024).

#### 3.2.2. The Role of PPI Domains in Cellular Processes and Infections

The significance of PPI domains is underscored by their governance over a vast array of cellular processes.

Through this exploration, we aim to highlight the indispensable role of PPI domains in the orchestration of cellular activities, in particular bacteriophage infection, and their potential implications in understanding and targeting various biological processes.

Receptor-binding proteins (RBPs), a subclass of PPI, play a crucial role in the specificity of bacteriophages, primarily determining their host range through interactions with various bacterial surface structures [123]. RBPs can be divided into two main classes based on their morphology: tail fibers and tailspike proteins (TSPs) [124]. Tail fibers are characterized by their long, slender, fibrous structure without enzymatic activity [125]. In contrast, TSPs are shorter, stockier, and typically possess enzymatic activity, often targeting specific surface structures like sugar moieties [125].

The interaction between bacteriophages and bacterial hosts is mediated by RBPs, which are the first point of contact. They bind to a range of structures displayed on the bacterial surface, including outer membrane proteins, lipopolysaccharides, capsular polysaccharides, and even organelles such as flagella or pili [126,127]. This interaction is a two-stage capture model, beginning with initial reversible binding, followed by more specific and irreversible binding to the receptors [128,129]. This process is essential for the phage’s infection process.

Furthermore, RBPs serve as the primary and most important checkpoint in the infection process [125]. These domains show significant sequence diversity, reflecting their specificity to host receptors and varying depending on the type of host receptor recognized and the infection process [123]. This diversity underscores the critical role of RBPs in mediating the specificity of bacteriophages to their bacterial hosts. In Gram-positive bacteria like *B. anthracis*, the cell wall is distinct in composition and structure. It lacks an outer membrane and features a thick peptidoglycan layer, transmembrane peptidoglycan-recognition proteins, and the nucleotide-binding oligomerization domain [130]. The transmembrane peptidoglycan-recognition proteins are potential phage receptors. For instance, the *B. anthracis* receptor for W*γ* phage has been identified as the LPXTG protein (a motif, known to be anchored by sortases to the bacterial peptidoglycan) GamR (gamma phage receptor) [131,132]. This protein’s role in virion binding, and the necessity of a potential secondary receptor for DNA delivery, highlights the complexity of phage–host interactions [132].

### 3.3. Fatty Acid-Binding Domains

In the following section, we turn our attention to fatty acid-binding domains, a minor but ubiquitous class of binding domains present across all organisms. After examining the two primary classes of binding domains that play pivotal roles in cellular processes, this segment aims to explore the significance and applications of fatty acid-binding domains. Notably, their potential in diagnostics, such as identifying structures composed of fatty acids like hydrophobic layers, highlights their importance despite being a less prominent class.

In cellular biology, fatty acid-binding domains (FABDs), such as the intestinal fatty acid binding domains (IFABPs) [68], are a key part of intracellular lipid-binding proteins. These domains are essential for identifying and attaching to fatty acids. The structure of fatty acid-binding proteins includes a β-barrel made up of 10 antiparallel β-sheets, which is topped by two short α-helical segments [68]. Proteins with these specific areas are known as intracellular lipid-binding proteins [133,134]. According to the literature, there is no existing classification for fatty acid-binding domains. However, classifications for fatty acid-binding proteins in human cells have been published based on the gene that expresses them (Table 2).

**Table 2:** List of fatty acid-binding proteins, based on the gene expression data from Smathers and Petersen [134].

Gene	Common Name	Aliases for Proteins	Localization
FABP 1	Liver FABP	L-FABP, hepatic FABP, Z-protein, heme-binding protein	Liver, intestine, pancreas, kidney, lung, stomach
FABP 2	Intestinal FABP	I-FABP, gut FABP (gFABP)	Intestine, liver
FABP 3	Heart FABP	H-FABP, O-FABP, mammary-derived growth inhibitor (MDGI)	Cardiac and skeletal muscle, brain, kidney, lung, stomach, testis, adrenal gland, mammary gland, placenta, ovary, brown adipose tissue
FABP 4	Adipocyte FABP	A-FABP, aP2	Adipocytes, macrophages, dendritic cells, skeletal muscle fibers
FABP 5	Epidermal FABP	E-FABP, keratinocyte-type FABP (KFABP), psoriasis-associated-FABP (PA-FABP)	Skin, tongue, adipocyte, macrophage, dendritic cells, mammary gland, brain, stomach, intestine, kidney, liver, lung, heart, skeletal, muscle, testis, retina, lens, spleen, placenta
FABP 6	Ileal FABP	Il-FABP, ileal lipid-binding protein (ILLBP), intestinal bile acid-binding protein (I-BABP), gastrophin	Ileum, ovary, adrenal gland, stomach
FABP 7	Brain FABP	B-FABP, brain lipid-binding protein (BLBP), MRG	Brain, central nervous system (CNS), glial cell, retina, mammary gland
FABP 8	Myelin FABP	M-FABP, peripheral myelin protein 2 (PMP2)	Peripheral nervous system, Schwann cells
FABP 9	Testis FABP	T-FABP, testis lipid-binding protein (TLBP), PERF, PERF 15	Testis, salivary gland, mammary gland
FABP 12	/	/	Retinoblastoma cell ^1^, retina (ganglion and inner nuclear layer cells) ^2^, testicular germ cells ^2^, cerebral cortex ^2^, kidney ^2^, epididymis ^2^

^1^ Expression found in humans, ^2^ expression found in rodents.

#### 3.3.1. Function and Specificity of FABDs

FABDs are key to the transport, storage, and metabolism of fatty acids within cells [135]. Their primary function is to bind long-chain fatty acids, enhancing their solubility in the aqueous environment of the cell and aiding their transportation to specific cellular sites. The affinity and specificity of these domains for particular fatty acids are influenced by the fatty acid’s saturation level. Generally, these domains show increased affinities for more hydrophobic molecules and decreased affinities for molecules with shorter chain lengths and higher unsaturation levels [134]. The binding affinity of FABDs is usually in the nanomolar range and varies depending on the chain length of the fatty acid [136]. While there is a high affinity for long-chain fatty acids, the affinity significantly drops (often >500 nM) for other hydrophobic ligands [136].

#### 3.3.2. Structural Characteristics of FABDs

The structure of fatty acid-binding domains (FABDs) is characterized by hydrophobic pockets that create an ideal environment for accommodating the fatty acid tail [137,138]. In tandem, specific amino acid residues within these domains engage the carboxyl head of the fatty acid, ensuring efficient and precise binding. This dual interaction plays a crucial role in the stability and functionality of FABDs.

Moreover, the stability of these interactions is enhanced by hydrogen bonds [139,140] and van der Waals forces [141]. These molecular forces not only stabilize the binding but also increase the affinity and specificity of the process. The amino acids are arranged in such a way that they often form a binding groove or cavity, tailored to fit specific fatty acids.

For example, in FAB-5—a subtype of FABDs—this tailored binding cavity is essential for its function, demonstrating the critical role of structural specificity. This specificity is vital for the biological functionality of FABDs, as it governs the selectivity for different fatty acids, influencing various cellular processes [141].

### 3.4. Carbohydrate-Binding Domains: Chitin-, Chitosan-, and Cellulose-Binding Domains

**Figure 4:**
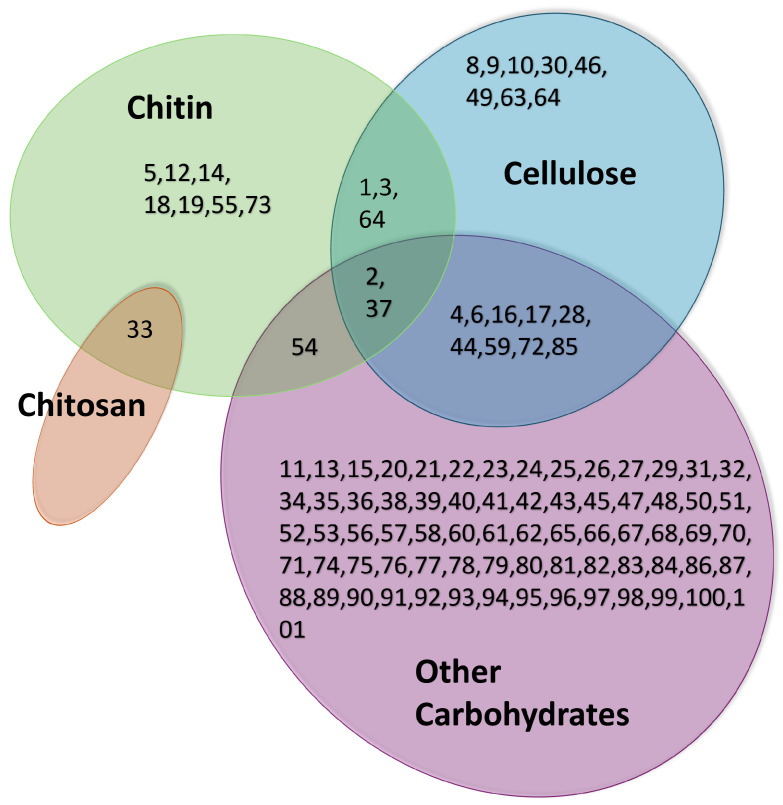
Visual representation of carbohydrate-binding domain families (CBMs) and their ligands according to data of [144] and modified. Differentiation is performed between amorphous and crystalline cellulose.

Carbohydrate-binding domains, also known as carbohydrate-binding modules (CBMs), are critical for specific binding to insoluble polysaccharides such as chitin, chitosan, and cellulose [142]. CBMs, naturally found in various organisms including Bacillus species and soil organisms, are integral to enzymes like chitinase. They function by reducing the distance between the substrate and the catalytic domain, thereby enhancing enzyme efficiency [142]. Usually, CBMs are classified based on amino acid similarities [69,143]. In the last 20 years, the number of families has increased from 39 to over 100. A further grouping into superfamilies has not been imposed yet [69]. However, Boraston et al. [69] further organized the CBM families into the following seven different groups based on structural similarities: β-sandwich, β-trefoil, cysteine knot, unique, OB fold, hevein fold, and unique (contains hevein-like fold). For the overview of our structure, we grouped the individual families according to the ligands chitosan, chitin, cellulose, and others; see Figure 4. For a more comprehensive breakdown of the classification based on the bound substrate, refer to the Appendix A section, specifically Table A1.

#### 3.4.1. Chitosan-Binding Domain

Chitosan has been widely studied for biomedical applications due to its biocompatibility and biodegradability. It is a derivate of the linear polysaccharide chitin. However, while chitin is composed of GlcNAc, chitosan is composed of GlcNAc and GlcN.

The chitosan-binding domain is a specific region within proteins or peptides that binds to chitosan, such as chitosanases [72]. Classified as carbohydrate-binding modules (CBMs), these domains are part of the carbohydrate-active enzymes, and their binding often depends on chitosan’s physical and chemical properties [72,145]. Chitosan-binding domains interact with chitosan through electrostatic interactions, hydrogen bonding, and hydrophobic effects. For example, since chitosan is amorphous, it is readily hydrolyzed by chitosanases. Chitosanases, however, do not act on chitin. The binding of chitosan-binding domains to chitosan, but not chitin due to acetylation, is facilitated by Van der Waals interactions and hydrophobic residues [145].

Furthermore, discoidin domains (DDs) in proteins, particularly those in CBM32 from *Dictyostelium discoideum*, demonstrate affinity for carbohydrates, including chitosan [146,147].

When combined with probes or markers, chitosan matrices can detect specific cancer cells or tumor microenvironments. This specificity can pave the way for developing diagnostic tools with higher accuracy and sensitivity [148].

#### 3.4.2. Chitin-Binding Domain

Chitin-binding domains (ChBDs) are a crucial component in enzymes that interact with carbohydrates. They bind catalytically active parts of the enzyme to a specific carbohydrate and concentrate them near the substrate [69,144].

Intein-mediated protein splicing is an application of ChBDs in recombinant protein purification [149,150]. The target protein is present as an N-extein to which an intein is bound. The ChBD is in turn fixed to this intein. The protein is isolated using a chitin affinity column by binding the ChBD to chitin in the column material. The thioester bond between the target protein and intein can be cleaved by adding higher concentrations of a free thiol via thiolysis. Higher temperatures also result in the release of the target protein [150]. The binding of the ChBD to chitin is mainly based on hydrophobic interactions between the aromatic side chains and the aliphatic regions in the pyranose ring of chitin [151]. The ChBD selectively binds to chitin and not to soluble derivatives of chitin or cellulose, as an antibody selectively binds to an antigen [75,152,153]. In general, different methods are known to characterize bindings. The binding affinity is defined as the tendency of two molecules to form a bond. The dissociation constant $K_{D}$, also known as the binding constant, is often used to describe this affinity. It reflects the balance between the dissociated and undissociated form and thus the average amount of binding; while high $K_{D}$ values (>$10^{-3}$ mol/L) indicate weak, unspecific binding, low $K_{D}$ values (<$10^{-10}$ mol/L) are a sign of very strong binding. Antigen–antibody bonds have binding constants in the nano- to micromolar ranges. The affinity also depends on the conditions in which the binding partners are present. The lower the affinity, the less specific the reaction of the antibody with the antigen.

The affinity of in *E. coli* recombinant synthesized ChBD from *B. circulans* indicates a dissociation constant of 149.72 ± 30.44 nM toward chitin of yeast cell bud scars [75]. Most $K_{D}$ values determined in the literature for proteins with bacterial ChBDs are 1–10 μM [76]. When determining the dissociation constant, a distinction is often made between α-chitin and β-chitin. For ChB proteins from *B. thuringiensis*, 3.460 ± 1.300 μM (β-chitin) and 5.250 ± 1.400 μM (α-chitin) were determined [154]. The same research group also determined ChBP values for ChBP derived from *B. licheniformis* with 4.120 ± 1.600 μM (β-chitin) and 5.980 ± 2.100 μM (α-chitin) [154]. A $K_{D}$ value of 1.400 ± 0.400 μM (*β*-chitin) was determined for the ChBP CBP21p from *Serratia marcescens* [155]. CBP21 is part of the chitinase B of *S. marcescens* [155].

#### 3.4.3. Cellulose-Binding Domain

Cellulose-binding domains (CBDs) are polypeptide bonds that belong to the subcategory of carbohydrate-binding modules. There are more and more modules being found in carbohydrate-active enzymes [156]. For this reason, these are also often investigated.

Cellulose-binding domains are generally found in cellulose-degrading enzymes such as cellulase [156]. Cellulase has a modular structure and is equipped with two domains. Most cellulases consist of a catalytic domain and a cellulose-binding domain, which are connected by a linker [157,158]. CBDs can occur both singly and repeatedly in these enzymes, with amino- or carboxy-terminal localization with respect to the catalytically active domain [157]. The catalytic domain contains the active center with the amino acid residues, which is responsible for the hydrolysis mechanism [158]. CBDs have highly conserved sequences with three aromatic residues. The binding of CBD to cellulose substrates is based on the interaction between the glucose rings of cellulose and aromatic amino acids, which are structurally located on the flat side of the domain [157,159].

CBDs mediate the adsorption of the enzyme to the substrate. This adsorption increases the concentration of the enzyme on the insoluble cellulose surface [157], which leads, for example, to an acceleration of enzyme-catalyzed hydrolysis [159].

To date, more than 180 different CBDs have been identified and categorized into more than 13 different protein families based on their amino acid sequence similarities. These can vary in size from 4 to 20 kDa and occur at different positions within the polypeptides: N-terminal, C-terminal, or internal [160]. Most CBDs belong to families I, II, and III [159]. Family I CBDs are compact polypeptides binding cellulose by three aromatic residues [161]. The CBDs of families II and III are much larger (and contain 90–100 and 130–172 residues), respectively [160]. They are specific for bacterial enzymes [160].

In addition to different structures, CBDs also have various properties. Some CBDs bind strongly to cellulose and can be used to immobilize active enzymes tightly [162]. Others bind reversibly and are better suited for separation and purification. Family I CBDs bind reversibly to crystalline cellulose and are a useful tag for affinity chromatography [163]. Interaction occurs through hydrogen bonding and van der Waals interaction [163]. They bind to cellulose in a pH range of 3.5 to 9.5, and the affinity of the tag is so strong that an immobilized fusion protein can only be released with buffers containing urea or guanidine hydrochloride. Thus, these denaturing elution conditions require refolding of the recombinant target protein [164]. In contrast, proteins with CBDs of families II and III can be eluted with ethylene glycol [164]. This is due to the low polarity of the solvent, which presumably interferes with the hydrophobic interaction at the binding site. Ethylene glycol can be easily removed by dialysis. In contrast to family I CBDs, family II CBDs can enhance the physical destruction of cellulosic fibers and release small particles from cotton fibers [165].

### 3.5. RNA-Binding Domains

RNA-binding domains (RBDs) are crucial regions within proteins, enabling specific recognition and binding to RNA molecules [166]. RNA-binding proteins (RBPs), a vast class of over 2000 proteins, ubiquitously interact with and regulate transcripts across various RNA-driven processes [167]. The central role of RNA in numerous cellular functions, from protein synthesis to gene regulation, underscores the importance of understanding RBDs and their interactions with RNA. This group of binding domains is categorized using various approaches, with the two most prevalent ones detailed in Table 3. These classifications are founded on distinctions among various domains.

Another method classifies RNA-binding proteins by the type of RNA that binds within their catalytic domains, according to Jahandide et al. [169]. The second approach for classification focuses on categorizing RNA-binding proteins based on the type of RNA they interact with. This methodology delineates specific groups depending on whether the proteins bind to 7S RNA, double-stranded (DS) RNA, messenger RNA (mRNA), or ribosomal RNA (rRNA). This system allows for a nuanced understanding of the functional diversity among RNA-binding proteins, emphasizing the significance of the RNA type engaged in the catalytic domain of these proteins.

#### 3.5.1. Structure and Function of RNA-Binding Proteins

RBDs engage with RNA through various interaction mechanisms, including hydrogen bonds, Van der Waals interactions, hydrophobic interactions, and π stacking interactions [170,171]. Statistical analysis reveals that approximately 23% of these contacts are potential hydrogen bonds, 72% are van der Waals interactions, and 5% are short contacts [170]. Specific binding typically arises from the combination of multiple RNA-binding regions along with additional weaker interactions with all parts of the RNA nucleotide.

The diverse RNA-binding protein family includes several notable subfamilies. The CUGBP Elav-like family (CELF) and muscleblind-like (MBNL) RBPs are instrumental in regulating alternative splicing and mRNA stability [172,173]. CELF proteins, comprising six members, have complex functions in both the nucleus and cytoplasm, influencing mRNA processing and stability [174]. Notably, CELF1 and CELF2 can function as tumor suppressors or oncogenes, depending on the cancer type [174]. Pharmacological targeting of CELF proteins, especially through organelle-specific drug delivery, presents new possibilities in cancer treatment [174].

#### 3.5.2. Important RNA-Binding Proteins in Therapeutic Applications

MBNL proteins, including MBNL1, MBNL2, and MBNL3, exert multifaceted control over gene expression. A study highlighting MBNL2’s role in tumorigenesis revealed its influence on cyclin-dependent kinase inhibitor 1A (p21CDKN1A) expression and DNA damage responses [175]. Manipulating MBNL2 levels impacts checkpoint kinase 1 (CHK1) phosphorylation, DNA repair, and cellular senescence, suggesting potential therapeutic avenues [170].

Generally, RNA-binding proteins can be classified by their binding mechanisms or their structural organization [176]. Around two-thirds of all studied mRNA-binding proteins are identified as having RNA recognition motif (RRM) domains. Within the MBNL family, zinc finger domains are recognized as superior [172]. Other important domains include DEAD-box helicase, KH domains, and cold shock domains, which are discussed in references [168,176,177].

AU-rich element RBPs (AU-RBPs) are another group of RNA-binding proteins with canonical and noncanonical functions. They are crucial in post-transcriptional gene regulation, particularly regarding DNA damage response and genomic stability. AU-RBPs like ZFP36 and AUF1 have implications in breast cancer [178]. Musashi proteins (MSI-1 and MSI-2), post-transcriptional regulators, are associated with cancer stem cell characteristics in ovarian cancer [179]. Strategies involving the dual knockdown of MSI1 and MSI2 show promise in ovarian cancer therapy [179].

Stress granules (SGs), cytosolic compartments formed under cellular stress, are emerging as important factors in liver diseases, including hepatocellular carcinoma (HCC) [180]. The RBP components of SGs are linked to HCC, highlighting their therapeutic potential.

Moreover, Kang et al. present various therapeutic strategies involving RNA-binding proteins, suggesting that detailed analyses of tumor molecular signatures could identify specific RBPs as targets in personalized cancer treatment [181].

R-loops, RNA/DNA hybrids, play dual roles in cells, affecting genomic stability and DNA damage responses [182]. Understanding the regulation of R-loops is vital for future therapeutic strategies, especially in cancer. For instance, Rad51, a factor in homologous recombination, is involved in R-loop formation, connecting RBPs to genomic stability [182,183].

In summary, RBPs operate in both the nucleus and cytoplasm, regulating RNA transcription and metabolism. Mutations in RBPs are associated with tumorigenesis, emphasizing their role in genomic stability. Future research may uncover the complex mechanisms by which RBPs control RNA/DNA hybrids, offering insights for treating cancer and other disorders [184].

### 3.6. Aptamers: The Nucleic Acid Antibodies

The last binding elements to be considered are aptamers. Aptamers are oligonucleotides, encompassing ribonucleic acid (RNA), single-strand deoxyribonucleic acid (ssDNA), or peptide molecules, characterized by their ability to bind to targets with high specificity and affinity. This binding capability arises from their unique three-dimensional structures [185]. Aptamers vary in length, typically ranging from 20 to 100 nucleotides. RNA and ssDNA aptamers, despite binding to the same targets, may differ in sequence and structural patterning [185,186]. As versatile biomaterials, aptamers have garnered attention in various fields, including biosensing, drug discovery, therapeutics, diagnostics, and drug delivery systems [187,188].

#### 3.6.1. Stability and Viability of Aptamers

Aptamers are composed of oligonucleotides, which exhibit greater thermal resistance compared to proteins, maintaining their structures through repeated cycles of denaturation and renaturation [189,190]. In contrast, proteins tend to denature and lose their tertiary structure at elevated temperatures [190]. This robustness at high temperatures provides a significant advantage for aptamers over protein-based antibodies, as aptamers can reanneal to regain their original shape and binding capability [191,192].

#### 3.6.2. Binding Mechanism

The binding mechanism of aptamers involves various forces, including van der Waals forces, hydrogen bonding, and electrostatic interactions [193,194,195]. Aptamers often exhibit a preference for positively charged sites in target proteins, as seen in complexes with NF-*κ*B, bacteriophage MS2 capsid, and lysin and arginin side chains [185,195]. However, exceptions exist, like the RNA aptamer targeting the human IgG1 Fc domain (hFc1), which binds despite the absence of positive charges on hFc1’s surface. It relies on weaker forces such as hydrogen bonds and hydrophobic contacts [196].

#### 3.6.3. Applications and Regulatory Milestones

Aptamers, particularly in oncology, offer potential in targeting cancer cells, tumor microenvironments, and molecules associated with tumor progression. They serve as both therapeutic agents and diagnostic tools because of the specific binding [197,198]. Optimizing aptamer sequences to improve binding affinity and specificity is crucial. This process can be achieved using the systematic evolution of ligands by exponential enrichment (SELEX) approach [185,199], which is reviewed by Kohlberger and Gadermeier [199].

Furthermore, aptamers have been investigated for targeting molecules associated with diseases like cancer or viral infection, such as adenovirus or SARS-CoV-2 [200]. A notable regulatory milestone was the FDA’s approval of pegaptanib, an aptamer targeting vascular endothelial growth factor (VEGF), for treating neovascular (wet) age-related macular degeneration (AMD) in 2004 [187].

## 4. Conclusions

The exploration of NBPs and aptamers offers a promising horizon for revolutionizing cancer therapy and diagnostics. Through their unparalleled specificity and affinity for target molecules, indicated in (Table 4), NBPs hold the potential to redefine precision medicine, enabling the development of highly effective, minimally invasive diagnostic tools and treatments. Despite significant advancements, the intricate mechanisms governing NBP interactions within the vast biological milieu remain partially understood, presenting a formidable barrier to their clinical adoption. Addressing this knowledge gap necessitates a multidisciplinary approach, integrating advanced bioinformatics, structural biology, and nanotechnology. As we delve deeper into the molecular intricacies of NBPs, the future of oncology and diagnostic medicine stands on the brink of a new era, promising more personalized, accurate, and effective healthcare solutions.

Ongoing research in this field is key to driving forward the evolution of cancer therapy. By delving deeper into the roles and functionalities of binding domains, there is a significant potential to transform cancer treatment paradigms and ultimately improve patient survival rates. This pursuit of knowledge in the realm of NBPs and their related domains is a crucial step towards a future where cancer treatment is more efficient, precise, and tailored to individual patient needs.

## Figures and Tables

**Table 1:** Classification of DNA-binding domains; adopted from Gonzales et al. [86] and modified according to Wingender [93].

Superclass	Class	Family
Basic domain		
	Leucine zipper factors	
		AP-1(-like) components
		CREB
		C/EBP-like factors
		bZIP/PAR
		Plant-G-box binding factors
		ZIP only
		Other bZIP factors
	Helix–loop–helix factors (bHLH)	
		Ubiquitous (class A) factors
		Myogenic transcription factors
		Achaete–scute
		Tal/Twist/Atonal/Hen
		Hairy
		Factors with PAS domain
		INO
		HLH domain only
		Other bHLH factors
	Helix–loop–helix/leucine zipper factors (bHLH-ZIP)	
		Ubiquitous bHLH-ZIP factors
		Cell-cycle controlling factors
	NF-1	
		NF-1
	RF-X	
		RF-X
	bHSH	
		AP-2
Zinc-coordinating DNA-binding domains		
	Cys4 zinc finger of nuclear receptor type	
		Cys4 zinc finger of nuclear receptor type
		Thyroid hormone receptor-like factors
	Diverse Cys4 zinc fingers	
		GATA factors
		Trithorax
		Other factors
	Cys2His2 zinc finger domain	
		Ubiquitous factors
		Developmental/cell cycle regulators
		Metabolic regulators in fungi
		Large factors with NF-6B-like binding properties
		Viral regulator
	Cys6 cysteine–zinc cluster	
		Metabolic regulators in fungi
	Zinc fingers of alternating composition	
		Cx7Hx8Cx4C zinc fingers
		Cx2Hx4Hx4C zinc fingers
Helix–turn–helix		
	Homeodomain	
		Homeodomain only
		POU domain factors
		Homeodomain with LIM region
		Homeodomain plus zinc finger motifs
	Paired box	
		Paired plus homeodomain
		Paired domain only
	Fork head/winged helix	
		Developmental regulators
		Tissue-specific regulators
		Cell-cycle controlling factors
		Other regulators
	Heat shock factors	
		HSF
	Tryptophan clusters	
		Myb
		Ets-type
		Interferon-regulating factors
	TEA domain	
		TEA
Beta scaffold factors with minor groove contacts		
	Rel homology region (RHR)	
		Rel/ankyrin
		Ankyrin only
		NF-AT
	STAT	
		STAT
	P53	
		P53
	MADS box	
		Regulators of differentiation
		Responders to external signals
		Metabolic regulators
	β-Barrel α-helix transcription factors	
		E2
	TATA-binding proteins	
		TBP
	HMG	
		SOX
		TCF-1
		HMG2-related
		UBF
		MATA
		Other HMG box factors
	Heteromeric CCAAT factors	
		Heteromeric CCAAT factors
	Grainyhead	
		Grainyhead
	Cold-shock domain factors	
		csd
	Runt	
		Runt
Other transcription factors		
	HMGI(Y)	
		HMGI(Y)
	Pocket domain	
		Rb
		CBP
	E1 A-like factors	
		E1A
	AP2/EREBP-related factors	
		AP2
		EREBP
		AP2/B3

**Table 3:** Classification of RNA-binding proteins based on their recognition surfaces, based on data of Lunde et al. [168] and modified.

Domain	Topology	RNA Recognition Surface Notes
RRM	αβ	Surface of β-sheet
KH type I	αβ	Hydrophobic cleft formed by variable loop between β2, β3, and GXXG loop
KH type II	αβ	Same as type I, except variable loop is between α2 and β2
dsRBD	αβ	Helix α1, N-terminal of helix α2, and loop between β1 and β2
Znf-CCHH	αβ	Primarily residues in α-helices
Znf-CCHH	Little regular secondary structure	Aromatic side chains form hydrophobic binding pockets for bases that make direct hydrogen bonds to protein backbone
S1	β	Core formed by two β-strands with contributions from surrounding loops
PAZ	αβ	Hydrophobic pocket formed by OB-like β-barrel and small αβ motif
PIWI	αβ	Highly conserved pocket, including a metal ion that is bound to the exposed C-terminal carboxylate
TRAP	β	Edges of β-sheets between each of the 11 subunits that form the entire protein structure
Pumilio	α	Two repeats combine to form binding pocket for individual bases, helix α2 provides specificity-determining residues
SAM	α	Hydrophobic cavity between three helices surrounded by an electropositive region

**Table 4:** Comparison of different protein classes and their corresponding binding affinities.

Protein Class	Specific Protein	Binding Affinity $K_{D}$ [nM]	Size [Amino Acids]	Reference
DNA-binding domain	DNA binding by glucocorticoid receptor	1.000		[201]
	DNA binding by androgen receptor	130		[201]
	DNA-binding proteins telomer repeat binding factor TRF1 and TRF2	200 and 750	63	[202]
	Prokaryotic transcriptional regulators of multiple antibiotic resistance in *E. coli*		129	[85]
Protein-binding domain	Competitive binding of a ligand to two receptors	100–80,000	Simulation data	[203]
	Spike protein and receptor-binding domain	314–3137		[204]
Fatty acid-binding protein	Human FABP1	127	17–23	[134]
Carbohydrate-binding domain	Chitin-binding domain of chitinase A1 from *Bacillus circulans*	149–228	45	[75,205]
	Chitin-binding domain of a lytic polysaccharide monooxygenase from *Cellvibrio japonicus*	2900–8500	58	[206]
	Chitin-binding domain from *Streptomyces*	110–2170	100/200/201	[207,208]
	Chitosan-binding module from *Paenibacillus elgii*		132	[209]
	Chitosan-binding module from *Paenibacillus sp.* 1K-5		260	[209]
	DD1	27,200–3,770,000		[146]
	*Clostridium cellulovorans* cellulose-binding protein A	500–1400	161	[210]
	Scaffoldin (CipA) containing a CBM3 family domain of Gram-positive bacterias such as *Clostridium thermocellulum*	400	150	[211,212]
	CBM4 glycanases from thermophilic and mesophilic bacteria	000– 0,000	150	[211,212]
	CBM10 families	4000 towards cellulose	45	[211,212]
	CBM14 from fungal tomato pathogen *Cladosporium fulvum* towards $\left(GlcNAc_{6}\right)$	6700	70	[211,213]
	CBM63 based on C-terminus of expansin BsEXLX1 from *Bacillus subtilis*	2100 towards cellulose	100	[211,214]
	CBM73 of trimodular LPMO	4300 towards α-chitin	60	[211,215,216]
	CBM86 of xylanase in *Roseburia intestinalis*	480,000 towards xylohexaose, 490,000 towards xylopentaose, 998,000 towards xylotetraose, and 1,900,000 towards xylotriose	138	[211,216]
	Cellobiohydrolase TrCel7A from *Trichoderma reesei*	2.9	36	[217,218]
	AD2 from *Fibrobacter succinogenes* S85	397.95	411	[219,220]
	AD4 from *Fibrobacter succinogenes* S85	838.51	207	[219,220]
RNA-binding domain	AGO2 let-7a	0.004–0.8		[221]
			90	[222]
Aptamer	JHIT-1–JHIT-7; LZH-1–LZH-17 against HepG2 target cells	3.9-2516.3		[223]
	Target: flavin mononucleotide	1100 ± 400		[224]
	Malachite green	950 ± 340		[224]

## Data Availability

No new experimental data were created or analyzed in this study. Data sharing is not applicable to this article.

## Appendix A

**Table A1:** Classification of carbohydrate-binding domains according to structural and functional properties [144]; however, to date, there remain over 6000 domains that are still unclassified [143].

Family	Protein Fold	Demonstrated Binding Specificities
CBM1	Cysteine knot	Cellulose (chitin one case)
CBM2	β-sandwich	Cellulose, chitin, xylan
CBM3	β-sandwich	Cellulose and chitin
CBM4	β-sandwich	Xylan, β-1,3-glucan, β-1,3-1,4-glucan, β-1,6-glucan, and amorphous cellulose
CBM5	Unique	Chitin
CBM6	β-sandwich	Amorphous cellulose, β-1,4-xylan, β-1,3-glucan, β-1,3-1,4-glucan, and β-1,4-glucan
CBM7	Deleted	
CBM8	Unknown	Cellulose
CBM9	β-sandwich	Cellulose
CBM10	OB fold	Cellulose
CBM11	β-sandwich	β-1,4-glucan and β-1,3-1,4-mixed-linked glucans
CBM12	Unique	Chitin
CBM13	β-trefoil	Mannose, xylan, N-acetylgalactosamine
CBM14	Unique	Chitin
CBM15	β-sandwich	Xylan and xylooligosaccharides
CBM16	β-sandwich	Cellulose and glucomannan
CBM17	β-sandwich	Amorphous cellulose, cellooligosaccharides, and derivatized cellulose
CBM18	Hevein fold	Chitin
CBM19	Unknown	Chitin
CBM20	β-sandwich	Granular starch, cyclodextrines
CBM21	β-sandwich	Starch
CBM22	β-sandwich	Xylan, β-1,3/β-1,4-glucans
CBM23	Unknown	Mannan
CBM24	Unknown	α-1,3-glucan
CBM25	β-sandwich	Starch
CBM26	β-sandwich	Starch
CBM27	β-sandwich	Mannan
CBM28	β-sandwich	Noncrystalline cellulose, cello-oligosaccharides, and β-(1,3)(1,4)-glucans
CBM29	β-sandwich	Mannan and glucomannan
CBM30	β-sandwich	Cellulose
CBM31	β-sandwich	β-1,3-xylan
CBM32	β-sandwich	Galactose, lactose, polygalacturonic acid, β-D-galactosyl-1,4-β-D-N-acetylglucosamine
CBM33	β-sandwich	Chitin and chitosan
CBM34	β-sandwich	Granular starch
CBM35	β-sandwich	4,5-deoxygalaturonic acid, glucuronic acid, xylan, β-galactan
CBM36	β-sandwich	Xylan and xylooligosaccharides
CBM37	Unknown	Xylan, chitin, microcrystalline and phosphoric acid-swollen cellulose, alfalfa cell walls, banana stem, and wheat straw
CBM38	Unknown	Inulin
CBM39	β-sandwich	β-1,3-glucan, lipopolysaccharide, and lipoteichoic acid
CBM40	β-sandwich	Sialic acid
CBM41	β-sandwich	Amylose, amylopectin, pullulan, and α-glucan oligosaccharide fragments
CBM42	β-trefoil	Arabinofuranose
CBM43	CtD-Ole e 9	β-1,3-glucan
CBM44	β-sandwich	Cellulose and xyloglucan
CBM45	Unknown	Starch
CBM46	Unknown	Cellulose
CBM47	β-sandwich	Fucose
CBM48	β-sandwich	Glycogen
CBM49	Unknown	Cellulose
CBM50	LysM-domain	Chitopentaose
CBM51	β-sandwich	Galactose and to blood group A/B-antigens
CBM52	Unknown	β-1,3-glucan
CBM53	Unknown	Starch
CBM54	Unknown	Xylan, yeast cell wall glucan, and chitin
CBM55	Unknown	Chitin
CBM56	Unknown	β-1,3-glucan
CBM57	β-sandwich	Glucose oligomers
CBM58	β-sandwich	Maltoheptaose
CBM59	β-sandwich	Mannan, xylan, and cellulose
CBM60	β-sandwich	Xylan
CBM61	β-sandwich	β-1,4-galactan
CBM62	β-sandwich	Galactose moieties found on xyloglucan, arabinogalactan, and galactomannan
CBM63	Expansin-like	Cellulose
CBM64	Unknown	Cellulose
CBM65	β-sandwich	β-glucan, xyloglucan
CBM66	β-sandwich	Fructans
CBM67	Multidomain structure	L-rhamnose
CBM68	Unknown	Maltotriose, maltotetraose
CBM69	Unknown	Starch
CBM70	β-sandwich	Hyaluronan
CBM71	β-sandwich	Lactose, LacNAc
CBM72	Unknown	Various polysaccharides, including cellulose, β-1,3/1,4-mixed linked glucans, xylan, and β-mannan
CBM73	β-sheet containing structure	Chitin
CBM74	Unknown	Starch
CBM75	Unknown	Xyloglucan
CBM76	Unknown	β-glucan, xyloglucan, glucomannan
CBM77	β-sandwich	Pectin
CBM78	β-sandwich	Decorated β-glucans, xyloglucan
CBM79	β-sandwich	β-glucans
CBM80	β-sandwich	Xylocglucan, glucomannan, galactomannan, barley β-glucan
CBM81	β-sandwich	β-1,4-, β-1,3-glucans, xyloglucan, avicel, cellooligosaccharides
CBM82	Unknown	Starch
CBM83	Unknown	Starch
CBM84	Unknown	Xanthan
CBM85	Unknown	Cellulose, glucuronoxylan, β-1,3-1,4-glucan, and glucomannan
CBM86	β-sandwich	Xylan
CBM87	α-β-α-domain	α-1,4-N-acetylgalactosamine-rich regions of galactosaminogalactan
CBM88	Unknown	Terminal galactose in galactoxyloglucan and galactomannan
CBM89	β-helix	Beechwood xylan and rye arabinoxylan binding
CBM90	Unknown	Ulvan
CBM91	Unknown	Xylans (birchwood and oat spelt)
CBM92	Unknown	β-1,3- and β-1,6-glucan
CBM93	Unknown	Glycan
CBM94	Unknown	N-Acetylglucosamine
CBM95	Unknown	Pectic rhamnogalacturonan-I
CBM96	Unknown	Alginate
CBM97	Unknown	Polygalacturonic acid
CBM98	Unknown	Amylopectin
CBM99	Unknown	Porphyran
CBM100	β-sandwich	Chondroitin sulfate
CBM101	β-sandwich	Agarose
